# Design of an ELC resonator-based reusable RF microfluidic sensor for blood glucose estimation

**DOI:** 10.1038/s41598-020-75716-z

**Published:** 2020-11-02

**Authors:** Greeshmaja Govind, M. Jaleel Akhtar

**Affiliations:** Department of Electrical Engineering, Indian Institute of Technology Kanpur, Kanpur, Uttar Pradesh 208016 India

**Keywords:** Lab-on-a-chip, Sensors and probes, Electrical and electronic engineering, Characterization and analytical techniques, Design, synthesis and processing

## Abstract

Design of a reusable microfluidic sensor for blood glucose estimation at microwave frequencies is presented. The sensing unit primarily comprises a complementary electric LC (CELC) resonator, which is made reusable by filling the test sample in a glass capillary before mounting it inside a groove cut in the central arm of the resonator. The use of glass capillary in the present situation to contain the blood sample actually eliminates the possibility of any direct contact of the sensor with the test sample, and hence wards off any coincidental contamination of the sensor. Usage of the capillary provides additional benefits as only microliters of the sample are required, besides offering sterile measuring environment since these capillaries are disposable. The capillary made of borosilicate glass is highly biocompatible and exhibits exceptionally high chemical resistance in corrosive environments. Apart from reusability, the novelty of the proposed sensor also lies in its enhanced sensitivity which is quite an essential factor when it comes to the measurement of glucose concentration in the human physiological range. The applicability of the proposed scheme for glucose sensing is demonstrated by performing RF measurements of aqueous glucose solutions and goat blood samples using the fabricated sensor.

## Introduction

Diabetes mellitus is a type of metabolic disease identified by hyperglycemia or the state in which the body cannot regulate the blood sugar level, developed due to defects in insulin secretion, action, or both^1^. The chronic disease is on the rise and has become one of the deadliest and the most expensive diseases to treat, even impacting the national economy, not to mention the hardship and distress it creates on the individuals and their families. Hyperglycemia, in the long term, leads to dysfunction and organ failure, especially of the brain, spinal cord, kidneys, eyes and heart. According to the International Diabetes Federation, by 2045, the number of diabetes patients would increase to an alarming number of 629 million, which would be a massive 10% of the global population^2,3^. It is presumed that one in two people go undiagnosed, and a significant share of the people with diabetes live in low or middle-income countries. However, if diagnosed in the early prediabetes stage, a combination of healthy lifestyle, and medications if one is at a high risk of diabetes, can prevent diabetes from progressing to type-2. Considering the gravity of the problem, the World Health Organization (WHO) in 2016 came up with the first global report on diabetes that provided significant leads towards understanding diabetes better^4^. The report calls for stronger responses from the society to best utilize science for developing affordable, cost-effective medical technologies for effectively addressing diabetes or in fact, to put a halt to the ever-rising disease. As an attempt to resolve this growing health challenge by devising cost-effective technologies for regular screening and early diagnosis, a microwave-based methodology for blood glucose estimation is proposed.

Glucose monitoring techniques have been in existence since the 1960s. Conventional glucose-sensing methods can be broadly classified into point sample methods and the more recent continuous monitoring methods^5^. The point sample testing is an enzymatic method done by means of finger pricking and tests on urine samples, whereas the continuous monitoring methods may be invasive, minimally invasive or non-invasive depending upon the use of intravenous/subcutaneous sensor^6^, microneedles^7,8^ or optical/transdermal sensors^9,10^, respectively. The proposed microwave-based measurement scheme is minimally invasive and provides ultrafast results and therefore is a good contender among continuous monitoring methods. Moreover, the use of microfluidic channels for holding the blood sample ensures that very minimal quantity of blood, merely in the order of microliters is required. The electromagnetic properties of the blood sample such as the permittivity and conductivity vary with the concentration of glucose, to which the microwaves are sensitive. By studying the variations in the reflection and transmission parameters of the designed sensor, the blood glucose levels may be estimated.

At microwave frequencies, blood glucose estimation has been carried out by various non-invasive and invasive methods. Non-invasive methods involve blood glucose estimation from the fingertips or earlobes using patch resonator, spiral microstrip resonator, ultra-wideband antenna, bandpass filter or spatially separated split ring resonators^11–15^. Even though non-invasive methods appear promising and convenient, they are associated with high degrees of unpredictability due to the variations in the skin thickness, applied pressure and fingerprints^16,17^. Invasive methods are, therefore, the more popular means of blood glucose monitoring at microwave frequencies. Various invasive sensing schemes using rectangular^18,19^ and cylindrical cavity sensors^20^, dielectric resonator^21^, band stop filter^22^, distributed microelectromechanical systems (MEMS) transmission line (DMTL)^23^, coaxial probes^24,25^, etc. are in use. Recently, microwave planar sensors based on metamaterial units such as split ring resonator (SRR)^26^, complementary split ring resonator (CSRR)^27^, open split ring resonator (OSRR)^28^, complementary electric LC (ELC) resonator^29^ and epsilon negative (ENG) unit cell resonator^30^ are gaining popularity for glucose sensing due to the enhanced sensitivity, low cost, compactness, and ease of manufacture and measurement.

The quantity of test sample adequate for observing acceptable sensor responses in the RF and microwave frequency range is very critical from the practical point of view. Earlier works have used Petri dishes, cuvettes and large containers for holding the sample but the necessity of having large quantities of blood and oversized sample holder limits their practical applicability^31–33^. Sample droplets may also be directly placed on to the sensor’s sensing region for clinical diagnostic applications^34,35^. Of late, microfluidic channels are extensively used for conveying the test sample to the sensing unit^36,37^. Such channels not only guide the samples efficiently to the sensing region but also the quantity of samples required is very less, almost as less as a few microliters or nanoliters. The microfluidic channels may be made of biocompatible materials such as glass^20,38^, silicon^19^ or polydimethylsiloxane (PDMS)^28–30,39^. Eventhough the state-of-the-art self-monitoring devices can work with blood sample volumes as low as 1 µl, majority of the meters, close to 71%, displayed incorrect readings when tested with this supposedly sufficient but still limited sample volume^40^.

Sensor reusability is another factor that must be deliberated while designing sensor systems. When the test sample comes in direct contact with the sensing region, even after flushing out, the sample leaves an imprint on the channel as well as the sensor that is capable of altering further measurements and the data fidelity^34,41^. If closed carriers such as glass or silicon capillaries are used for carrying the test sample, the error that would have arisen due to the remnants left on the sensing regions could be obviated. Even so, the conventional microfluidic channels would still bear the history of the previous samples and interfere with the measurement process. The proposed sensing scheme thus puts forward the novel idea of using disposable glass capillaries in lieu of conventional microfluidic channels for ensuring error-free and faithful data acquisition, besides guaranteeing sterilized measuring environment and ease of handling. Considering the commercial aspect, even though the state-of-the-art glucose sensors have moderately low fixed costs, their variable costs in the form of lancets and test strips are exceedingly high, making them unaffordable for a long term use. Whereas, in the case of microwave sensors, despite the fixed costs being comparable to that of the commercial glucometers, the variable costs, incurred for the disposable glass capillaries, are negligible, making them a very economical alternative. Another factor that has encouraged to seek an alternative to the modern-day glucose monitoring devices is their limited shelf life. The glucose sensing strips work on complex biochemical reactions and consequently, the finite lifetime of chemicals may lead to strip failures. The proposed scheme using the microwave resonators is devoid of any biochemical treatments and hence there are no major factors that would limit the lifetime of these sensors.

There are quite a few other factors that affect the accuracy of the state-of-the-art glucose sensors and the proposed microwave microfluidic sensors equally; the most important of them being the patient factors such as blood sample composition and pharmacologic state. Almost every state-of-the-art blood glucose monitoring system measures the glucose concentration of a complex composition of blood, which is then calibrated against plasma glucose. Diversity in the cellular, molecular and salt content of the sample, therefore, has an effect on the measurement. An approach to implement the proposed microwave sensing modality free from the inaccuracies due to composition, to a certain extent, would be to use plasma samples in place of whole blood samples. However, this could be a viable alternative in a laboratory testing environment, but inexpedient for a self-monitoring device, which is the ultimate design intent of the proposed sensor. Hematocrit levels may interfere with the readings of a glucometer as the glucose content in the cells are different from that of the plasma^42^. In the commercially available glucometers the blood cells can alter the electron flow or the enzymatic reactions. This situation does not occur in glucose sensing with the proposed sensor. Certain substances in the blood that occur naturally or present during diseased states such as triglycerides, oxygen, uric acid, acetaminophen and ascorbic acid are found to affect the accuracy of electrochemical blood glucose monitoring systems due to the way they react with the mediator enzymes and electrodes. These factors *prima facie* do not interfere with the proposed measurement procedure using microwave sensors, but neither the proposed sensor nor any state-of-the-art glucometers have the capability to infer whether a wrong meter reading was caused because of these factors.

In this context, the design of a reusable microfluidic sensor for monitoring blood glucose at microwave frequencies is presented. The sensor is inspired by the metamaterial structure of complementary electric LC resonator. The central arm of the resonator is modified to form a cavity by carving in a groove deep into the substrate and then coating metal on the sidewalls for enhancing the capacitance. The test sample, collected in disposable Borosilicate glass capillary, is then placed into the cavity and the responses are observed. With the exception of a handful of work on human serum^34,43^ and pig blood samples^19,31,32^, a vast majority of the studies in the research area of microwave-assisted glucose sensing relies on using aqueous glucose solutions as the test sample due to the intricacies in using real blood samples for the experimental validation. In this work, aqueous solutions of glucose, as well as blood samples from goat, are used for the study. The dependency of the glucose concentrations on the sensor’s response could be translated to predictable relationships with the help of mathematical models based on the resonant frequency shifts^44^. The proposed measuring strategy may be endorsed as a primary screening method for blood glucose monitoring.

## Design procedures

### Design of microfluidic channel

While dealing with practical measuring scenario, blood glucose estimation requires specific precautionary measures to be followed for improving the accuracy^41^. Careful handling of the samples and preventing their exposure to contaminated environment is the foremost of all. Secondly, a major detrimental factor that limits the usage of microwave planar sensors for glucose sensing is the inability to ensure independent measurements. There are two predicaments that have to be dealt with when reusing the same equipment for the measurement. First, is associated with the reusability of the sample holder, i.e., the microfluidic channels conveying the glucose or blood samples have to be meticulously cleaned or sterilized after each measurement as the sample leaves its signature in the form of remnants in the tract/passage walls which affect further measurements. Second, is associated with the sensor; since the sensor comes in direct contact with the test sample, the sensor too has to undergo frequent sterilization procedures. In this scenario, the use of disposable sample holders is suggested, thus ensuring a sterile environment for testing, keeping the channel and sensor contaminations at bay. Capillaries made of laboratory-grade Borosilicate glass, having length 30 mm, outer diameter 2.2 mm, thickness 0.2 mm, and approximate maximum capacity of 95 µl, are used as sample holders in this work. They can be sealed from both the ends after filling the sample, thus isolating from further external contact. Hence the use of disposable capillaries eliminates the need for sterilizing the holder and the sensor, and expedites the measurement process. Furthermore, it is non-viable to use large volumes of blood even if it means improved sensor performances with more sample volume to interact with. This is another reason for endorsing the use of microfluidic capillaries. Due to their availability in sterilized state, convenience and cost-effectiveness, disposable sample holders may be envisioned as a solution of the future, fast replacing the conventional microfluidic channels used in microwave sensing schemes.

### Sensor design and sensitivity analysis

The sensor comprises a transmission line and a resonator etched on the ground plane. The test sample is placed on top of the resonator structure, which interacts with the electric field coupled from the host line. The intensity of the electric field in the sensing region of the resonator plays a paramount role in determining the sensitivity of such planar sensors. The proposed sensor design has a CELC resonator in conjunction with an incised groove as the basic sensing unit. The sensor is designed progressively from a conventional rectangular complementary split ring resonator (CSRR). It is a well-established fact that the strength of the electric field appears the highest in the arm of the CSRR, opposite to the resonator gap^45^. The test sample, therefore, has to be suitably placed in the arm opposite to the resonator gap for maximum field coupling. Thus, with the objective of maximizing the sensitivity, starting from the basic conventional rectangular CSRR based sensor, the evolution of the proposed sensor design is presented in this section. The sensors are designed on an 80 mm wide and 100 mm long Rogers RT5880 substrate of thickness 3.175 mm, relative permittivity, $\varepsilon_{r} = 2.2$ and loss tangent, $\tan \delta = 0.0009$. The copper metallizations are 35 microns thick, and the 50 Ω transmission line is 9.6 mm broad. The full-wave simulations are carried out in CST Microwave Studio.

The sensitivity may be increased forthrightly by reducing the CSRR structure dimension; however, this strategy cannot be implemented in this design due to the size restrictions. Decreasing the width of the sample holding arm of the CSRR structure increases the gap capacitance and hence the intensity of the electric field, but cannot be reduced lesser than 3 mm as the capillary has to be placed there. Likewise, the length of the CSRR structure cannot be reduced lower than 30 mm, a constraint imposed by the capillary length. Thus in all the designs to follow, the optimizations on the dimensions are carried out within these specified limits.

### Design 1: modified rectangular CSRR

**Figure 1:**
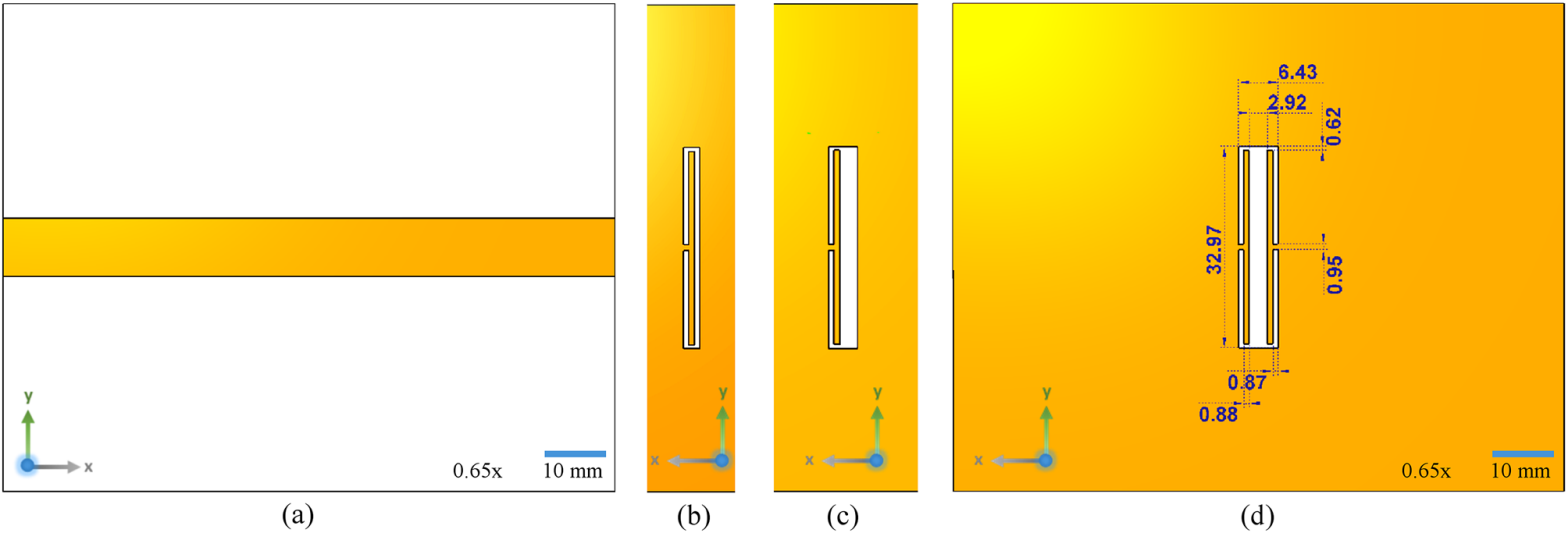
(**a**) Top view of the sensor showing the transmission line and (**b**) the conventional CSRR structure etched on the ground plane. (**c**) Design-1, a modified CSRR structure. (**d**) Design-2, a complementary ELC resonator on the ground plane of the sensor along with the design parameters. The scale bars are also indicated alongside.

Figure 1a illustrates the top view of the sensor, showing the microstrip line, and Fig. 1b shows the bottom ground plane of the sensor with the etched out CSRR structure. The resonator design of Fig. 1b, which is a conventional rectangular CSRR, is slightly transformed so as to have one broader arm where the sample under test is intended to be placed. The modified sensor is shown in Fig. 1c. The sensor resonates at a frequency of 2.6 GHz. The active sensing region, i.e., the broader arm of the sensor, is 2.92 mm wide and 32.97 mm long for comfortably placing the sample.

### Design 2: CELC resonator

In order to increase the electric field concentration in the broader arm, intuitively, the same CSRR design may be superimposed with its mirrored counterpart, giving rise to the CELC resonator of Fig. 1d. The sensor has a resonant frequency of 2.31 GHz. Although the sensor is more sensitive than the previous design due to the enhancement in the average field intensity, the design needs further improvement since the capillary has a circular cross-section and hence the sensor would be loaded at just one and only one point which does not allow for sufficient field interaction. Rectangular capillaries may be employed as an alternative; however, the difficulty involved in custom manufacturing such capillaries of diminutive sizes would ensue additional cost.

### Design 3: CELC resonator with embedded cavity and metalized sidewalls

**Figure 2:**
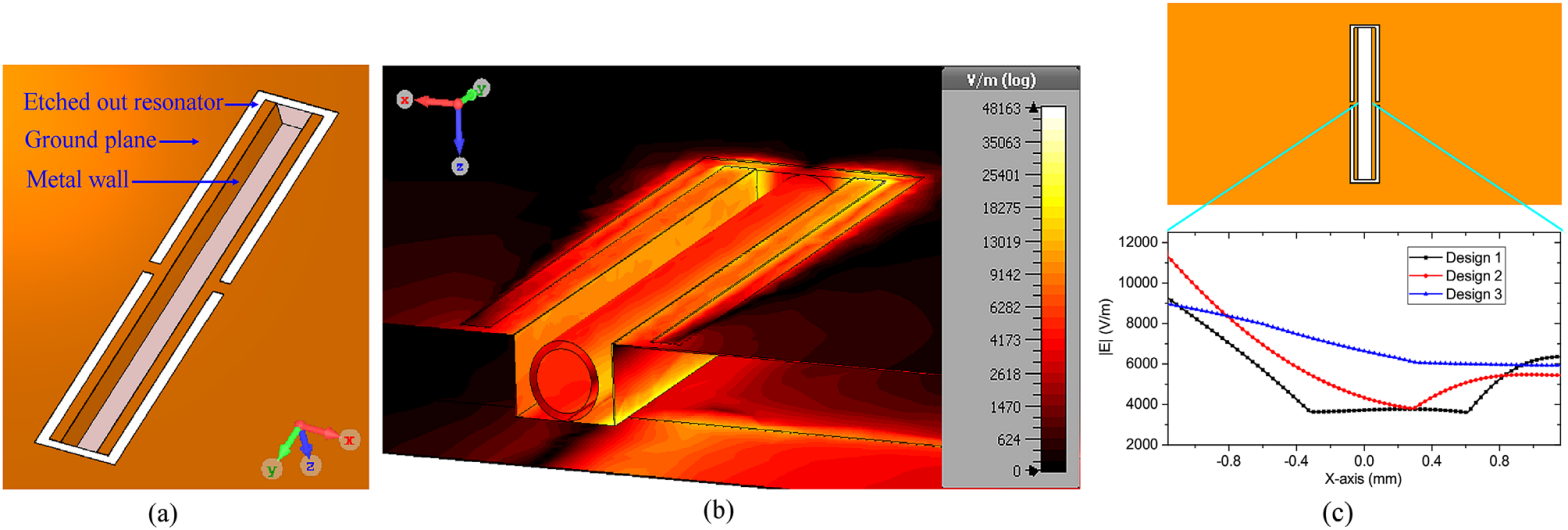
(**a**) Sensor with metallized groove sidewalls. (**b**) E-field distribution at the resonant frequency of 1.9 GHz with the glass capillary placed in the sensing region. (**c**) E-field intensity along the length of the sensor, through the centre of the cavity, and at the position equal to half the sidewall depth from the surface.

The CELC resonator has improved sensing capability compared to the conventional CSRR structure. Nonetheless, the constraint in reducing the width of the sensing arm limits any further attempt to intensify the electric field strength and hence the sensing capability beyond the achieved standards. Alternatively, the sensing arm may be visualized as a parallel plate capacitor of plate area defined by the length of the central arm (31.73 mm), width given by the thickness of copper metallization (35 µm); and plate separation equal to the width of the arm (2.92 mm). The capacitance of the parallel plates and consequently, the field concentration may be amplified by increasing the plate area alone, as there is limitation in reducing the plate separation, as discussed earlier. The plate area is therefore increased, as shown in Fig. 2a, by extending the ground plane as the sidewalls of a 2 mm deep groove^46^. The groove is made by carving out the substrate in the central arm of the CELC structure. Figure 2b shows the electric field intensity distribution at the sensor’s resonant frequency of 1.9 GHz; the glass capillary containing the sample, placed in the sensing region can also be seen. The glass capillary is modeled as a Pyrex glass container of relative permittivity 4.82 and loss tangent 0.0054.

A comparative plot of the electric field intensity in the active sensing region of all the discussed designs is presented in Fig. 2c. The abscissa shows the distance along the length of the sensor with the midpoint of the sensing region as the origin. For all the designs, the field is calculated at the center point of the sensing region, i.e., at $y = 0$, along the plane that passes through the face center of the sidewalls, i.e., at a depth of $z = 0.0175{\text{ mm}}$ for designs 1 and 2, and $z = 1{\text{ mm}}$ for design 3, measured from the surface of the sensor. As evident from the electric field distributions of the figure, design 3 has the densest concentration of electric field, i.e., 6634 V/m, at the center of the sensing region ($x = 0$) as compared to the previous configurations of design 1 and design 2, having field intensities of 3720 V/m and 4436 V/m, respectively. Also, in design 3, the field intensity is reasonably uniform throughout the sensing region.

**Figure 3:**
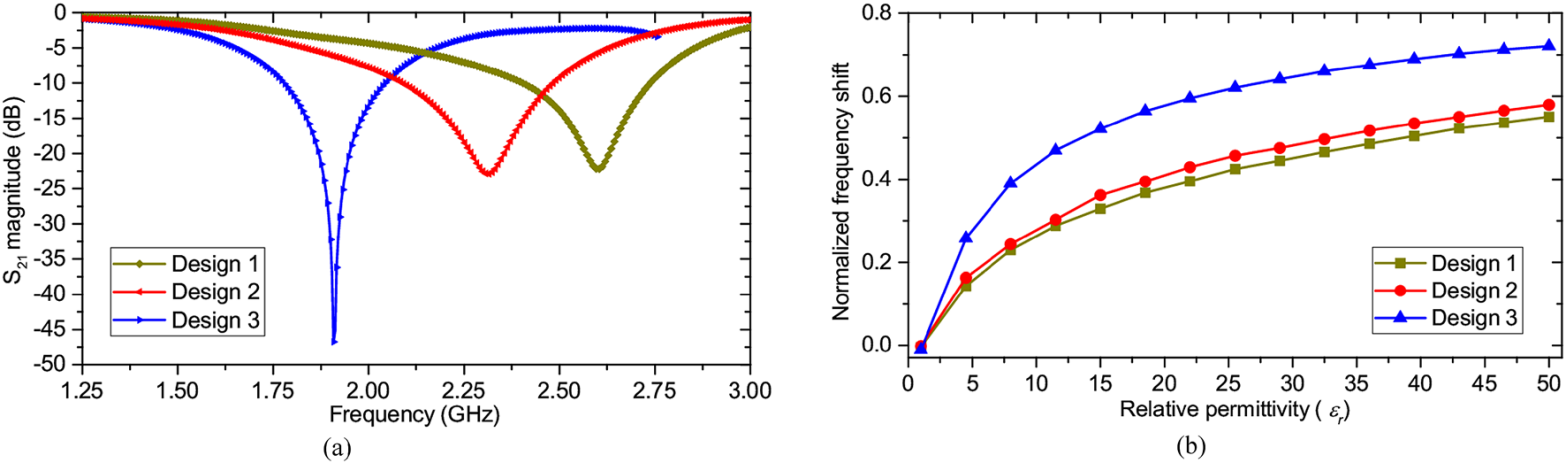
(**a**) Resonant frequencies of different sensor configurations. (**b**) Comparative plot showing the normalized frequency shifts produced by the sensors for samples with varying permittivity.

To analyze the sensitivity across all the sensor variants, the relative permittivity, $\varepsilon_{r}$, of a lossless test sample placed over the central arm of the resonator, is varied over a wide range and the resultant loaded resonant frequency, $f_{r}$, is observed. The normalized frequency shifts, ${{\left( {f_{0} - f_{r} } \right)} \mathord{\left/ {\vphantom {{\left( {f_{0} - f_{r} } \right)} {f_{0} }}} \right. \kern-\nulldelimiterspace} {f_{0} }}$, where $f_{0}$ is the sensor’s unloaded resonant frequency, are then compared to ensure a fair assessment as it is known that a sensor’s higher resonant frequency in itself could be a partial contributing factor towards achieving more significant frequency shift and thus higher sensitivity^47,48^. Figure 3a shows the unloaded resonant frequencies of the sensors. The proposed sensor design, design-3, possesses exceptionally high Q-factor of 329.23 as opposed to design-1 and design-2, having Q-factors equal to 34.94 and 26.06, respectively; which makes the proposed design-3 extremely suitable for characterizing low loss samples. The normalized frequency shifts plotted as a function of the test sample’s relative permittivity, corresponding to all the sensor designs are shown in Fig. 3b. Figure 3b clearly demonstrates the superior sensing capabilities of the newly proposed design, compared to the conventional CSRR and CELC designs, which could be attributed to the higher field concentration, as evident from Fig. 2c. Consequently, design 3 is finally chosen to carry out the experiments.

## Measurement and results

**Figure 4:**
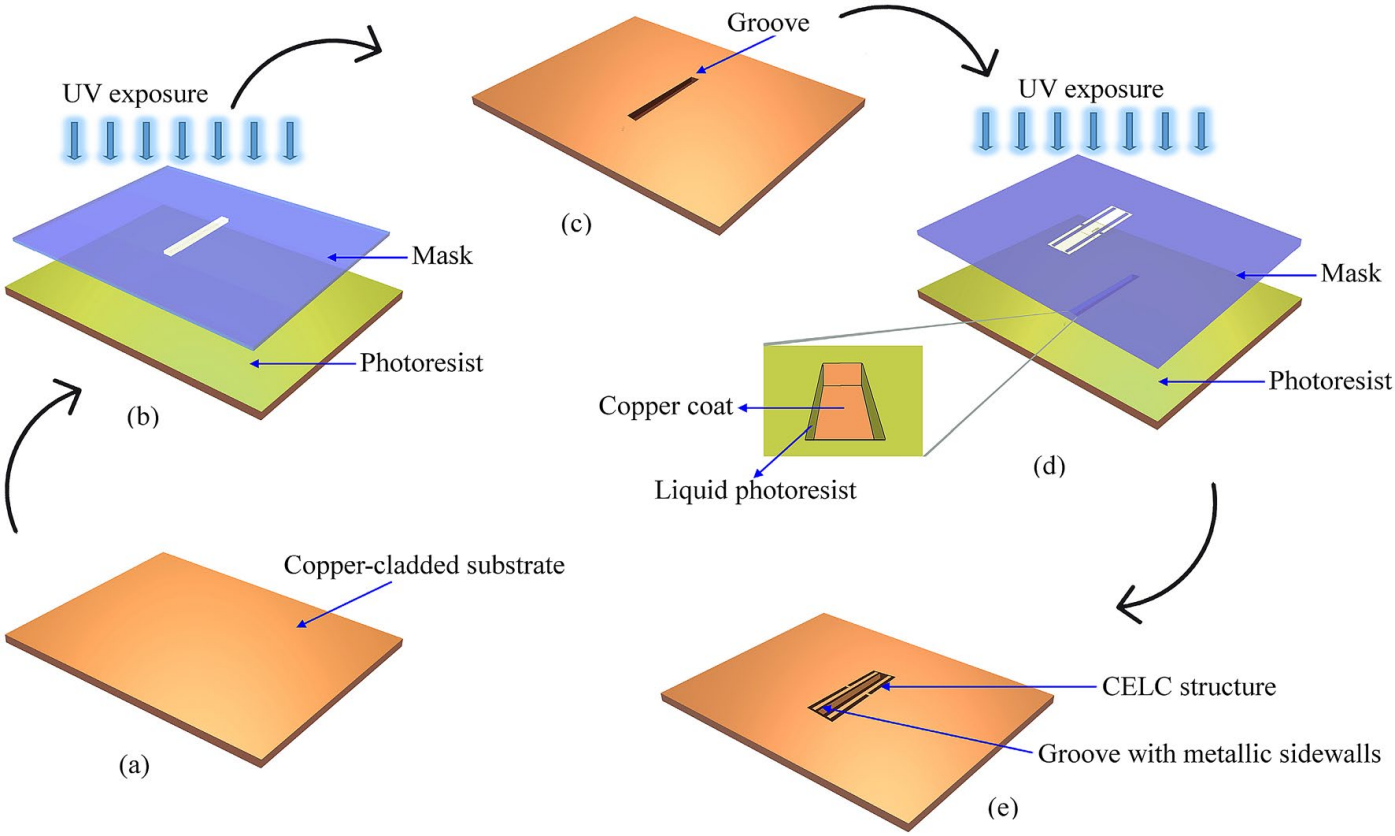
Illustration of the sequential processes involved in the sensor fabrication. (**a**) Rogers RT5880 substrate. (**b**) Substrate coated with photoresist, exposed to UV source through a mask to transfer the location of the groove. (**c**) Substrate with a groove milled into it. Next, the surface is copper plated. (**d**) Substrate with copper plated groove is coated with photoresist and exposed to UV source through a mask for etching the CELC resonator structure. In the inset, the liquid photoresist coated sidewalls are shown. (**e**) Final configuration showing the embedded groove and metallized sidewalls.

The designed sensor is fabricated and its applicability in differentiating samples of different glucose concentrations is studied. The photolithographic fabrication techniques adopted in realizing the proposed sensor are illustrated in Fig. 4. In this process, initially, the masks are generated by transferring the pattern onto a photographic film (Fujifilm plotter film HG XPR-7S) in a photoplotter and then developed manually in a tray using Fujifilm QR-D1 developer. At this stage, the film is inspected for defects such as track breakages or short circuits. Meanwhile, the entire substrate, shown in Fig. 4a, is uniformly laminated with a negative photoresist by placing the substrate in between heated feed rollers. The laminated substrate is prebaked at a temperature of 100–120 °C for 10 min in a convection oven to densify the photoresist by vaporizing the coating solvent. After careful alignment of the masks on both sides, the laminated substrate is exposed to ultraviolet (UV) light in a double‐sided drawer exposure unit with a vacuum system for transferring the pattern, as shown in Fig. 4b. As a result, the portion of the laminate that is not protected by the mask becomes etch-resistant. The substrate is treated with a developer in a spray developing unit where the unexposed part gets dissolved in the developer. The photoresist developer used is sodium carbonate. The entire laminate surface is developed simultaneously with the help of a rotary system present inside a developing chamber. After developing, the copper from the portion of the laminate protected from UV is etched away using ferric chloride copper etchant solution, by hanging it in a foam etching center. The substrate is now ready with the desired pattern, but the photoresist that is remaining on the substrate needs to be stripped away. The stripping is carried out in a stripping cuvette in which the remains of the photoresist are removed by rinsing the substrate in sodium hydroxide solution. Finally, the substrate is dried by placing it in an oven.

In order to realize the sensor, firstly, a rectangular patch of dimensions 2.92 × 31.73 mm^2^ is etched on top of the Rogers RT5880 substrate that would help in identifying the location where the groove has to be constructed. A rectangular pocket of depth of 2 mm is then cut at this site by removing the substrate using a three-axis high precision CNC milling machine. The substrate with the sensing cavity is shown in Fig. 4c. Next, the residual copper coating of the structure of Fig. 4c is removed and copper-plated anew so as to have a copper layer of uniform thickness (35 µm) all over the substrate, including the interior of the groove. The process described in Fig. 4b is now repeated, this time, with a new mask, having the pattern of the CELC resonator of Fig. 1d. In advance to the UV exposure, the longer faces of the groove are coated with liquid photoresist so that the copper cladding remains only on these faces that form the sidewalls of the groove, after exposure and development using sodium carbonate. The process is illustrated in Fig. 4d; the liquid photoresist coated sidewalls can be seen in the inset figure. The sensor prototype with embedded cavity and metalized sidewalls is shown in Fig. 4e. Subsequently, the mask with the geometry of the microstrip line of Fig. 1a, is then constructed on the top plane to form the complete sensor. Particular attention has to be paid while attaching the SMA connector (part number: R124 403 123, 14.43 long and 12.7 mm wide) with an inner conductor diameter of 1.27 mm onto the 9.6 mm broad microstrip line as there is a high probability of the outer conductor coming in contact with the microstrip line. Thus to stay clear of this uncertainty, the sensor is configured with an allowance of 0.25 mm at the shorter ends of the sensor, where the copper is stripped off the substrate so that the SMA outer conductor and the microstrip line are separated in space.

**Figure 5:**
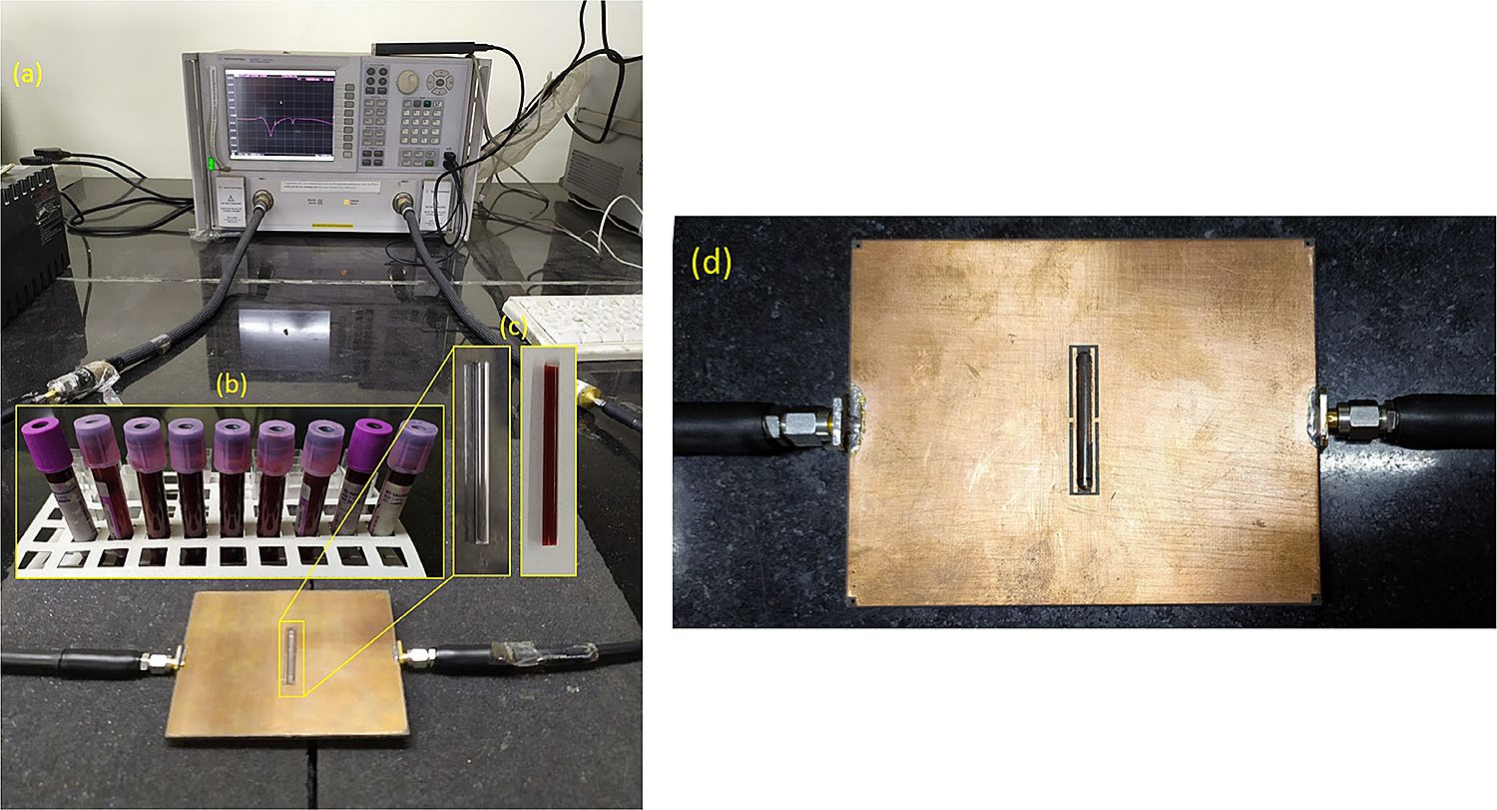
(**a**) Experimental set up showing the sensor connected to the VNA. (**b**) Goat blood samples of different concentrations, collected in K3-EDTA tubes. (**c**) Empty and blood-filled borosilicate glass capillaries used for testing. (**d**) The sensor prototype with the sample filled microfluidic channel placed in the sensing region.

The experiments are performed using Agilent N5230C vector network analyzer (VNA) in the frequency range of 1–5 GHz. Figure 5a illustrates the fabricated sensor prototype, connected to the VNA. The samples are prepared from freshly collected goat blood from slaughterhouse in purple/lavender-cap BD Vacutainer® spray-coated K3-EDTA tubes that are typically used for whole blood hematological studies. The blood samples in EDTA tubes are shown in Fig. 5b. The samples may be preserved in a refrigerator for up to three days. A controlled amount of d-Glucose is then added to the blood specimens, and samples having concentrations of 100 mg/dl, 200 mg/dl, 300 mg/dl, 400 mg/dl and 500 mg/dl are prepared. The initial glucose concentration of goat blood was taken into consideration while preparing the samples. The reference sugar level of the goat blood was 88 mg/dl, as measured using OneTouch® SelectSimple™ blood glucose monitoring system. In addition to the blood samples, aqueous glucose solutions are also prepared using d-glucose and deionized water. The samples are then injected into the custom-manufactured Borosilicate glass capillaries, shown in Fig. 5c using a plastic medical syringe. As can be seen from the figure, the ends of the capillary need not necessarily be sealed to prevent the sample from flowing out; instead, the sample stays in place due to the surface tension alone. However, the ends of the capillary may be sealed to prevent external contamination. The sample holders are disposable and may be discarded after use, to ensure that the current data acquisition is not affected by the residual traces of previous samples.

**Figure 6:**
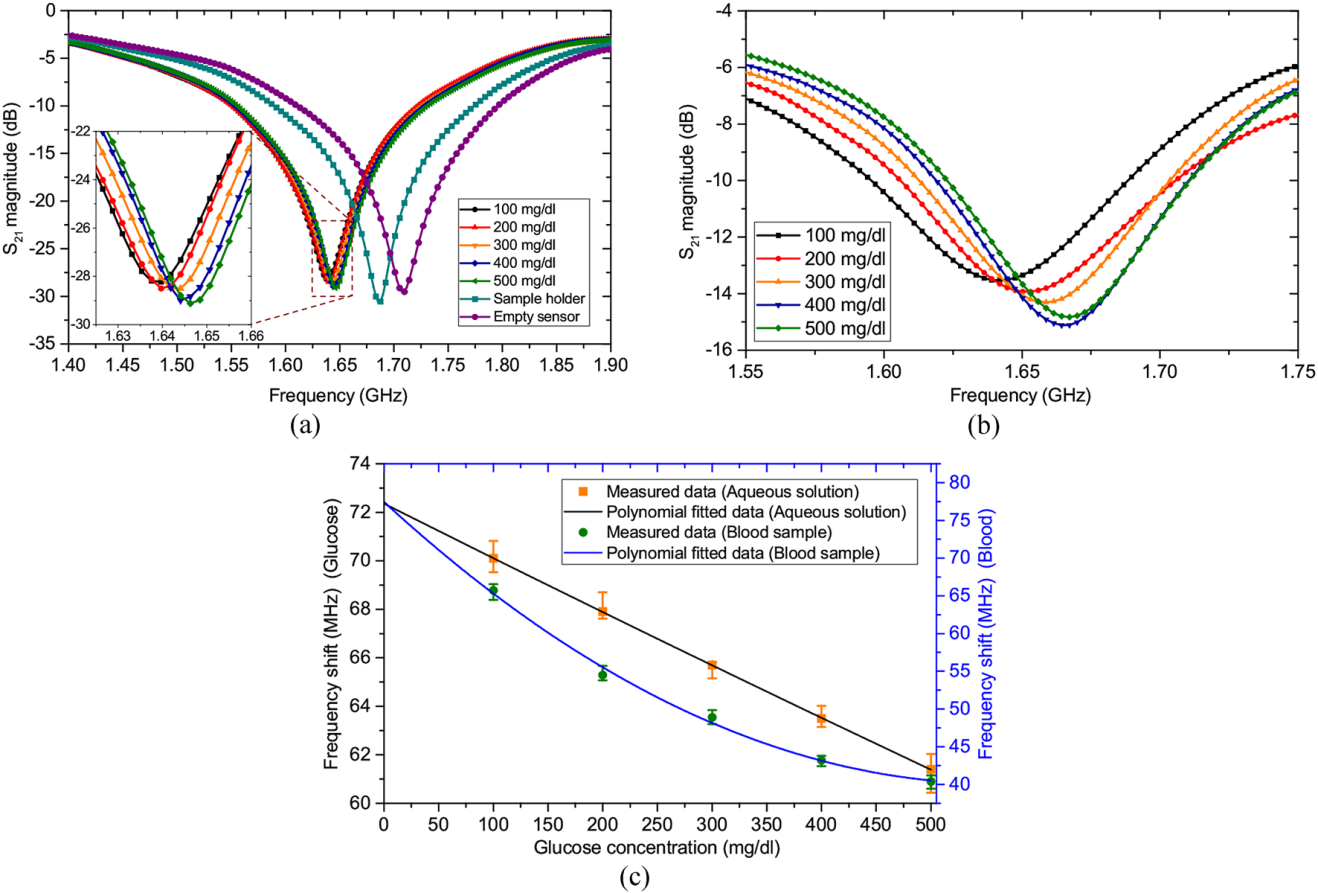
Measured transmission parameters when the samples under test are (**a**) aqueous glucose solution and (**b**) goat blood. (**c**) Measured and curve-fitted frequency shifts of the aqueous glucose and goat blood samples.

The measured results of aqueous glucose and blood samples are presented in Fig. 6. The measured resonant frequency of the sensor in the unloaded condition is 1.71 GHz, deviating slightly from the simulated value, as the metallization and fabrications of the delicate, narrow and shallow components do not match perfectly with the simulated model. However, this deviation does not interfere with the characterization of samples as each resonator is calibrated and modelled based on a measured dataset^45^. Two aspects have to be recognized while monitoring glucose using microwave sensors. First, the glucose concentrations of the test samples do not have a linear correlation with their dielectric properties. Second, the relationship of the transmission responses of the sensor with the samples’ dielectric properties is also are not linear. This varies from sensor to sensor and is unique^34^. It is in accordance with this relationship that a sensor is calibrated so that the readings shown are based entirely on the frequency shift of that particular sensor alone. When the empty glass container is placed in the sensing region, the resonant frequency is found to have shifted to 1.6856 GHz. Figures 6a and b, respectively, show the measured transmission responses when the samples under test are aqueous glucose solution and goat blood. While testing the samples of aqueous glucose solution, the sensor is observed to have a sensitivity of 0.0185 MHz/mg dl^−1^. On the other hand, when the blood samples are tested, the sensor is found to be more sensitive, having a sensitivity of 0.056 MHz/mg dl^−1^. This reduced sensitivity is anticipated in the case of aqueous samples as the sensor’s sensitivity tends to saturate at higher permittivity levels, as evident from the plot of Fig. 3b. It is well-known that the blood samples have much lower dielectric constant compared to the aqueous solutions, and hence the better distinguishability. The glucose concentrations and the deviations in the frequencies from the unloaded resonant frequency show good quadratic correlation; the curve-fitted plots are presented in Fig. 6c, for both the aqueous and goat blood samples. Each sample was measured six times and the deviations are shown by the non-overlapping error bars for each concentration. The regression equations are given by (1) and (2) for the aqueous glucose and blood samples with the goodness of fit calculated as $R^{2} = 1$ and $R^{2} = 0.9955$, respectively.

1 $$ \Delta f_{a} = 1.429 \times 10^{ - 6} g^{2} - 2.266 \times 10^{ - 2} g + 72.36 $$

2 $$ \Delta f_{b} = 1.193 \times 10^{ - 4} g^{2} - 0.1335g + 77.46 $$

where $\Delta f_{a}$ and $\Delta f_{b}$ are the measured frequency shifts in MHz corresponding to the aqueous glucose and blood samples, respectively, for a glucose concentration of $g$ mg dl^−1^. The measurements are carried out at room temperature of 23 °C. Though temperature is one of the many physical factors that can influence the measurement readings, the ambient temperature variations have had hardly any impact unless the conditions are extreme. Likewise, the sensor performance was not found to have any notable variation with time.

The proposed sensor is compared with several glucose-monitoring microfluidic sensors that have been reported earlier. The studies are presented in Table 1, in the increasing order of the sensitivities. The proposed sensor exhibits improved performance compared to the previous works, besides having the benefit of being reusable. It has to be noted that the test liquids in all of these works are aqueous glucose solutions.

**Table 1:** Performance comparison of glucose-monitoring microfluidic sensors.

References	Technique	Reusability	Sensitivity (MHz/mgdl^-1^)
Reference^18^	Rectangular waveguide cavity	No	4.00E−04
Reference^28^	Open split ring resonator (OSRR)	No	~ 1.88E−03
Reference^48^	Circular CSIW resonator	No	3.83E−03
Reference^30^	ENG unit-cell resonator	No	1.00E−02
Reference^23^	Distributed MEMS transmission lines (DMTL)	No	1.64E−02
This work	CELC resonator with groove	Yes	1.85E−02

## Conclusion

In this work, the design of a reusable microwave microfluidic sensor for monitoring blood glucose concentration is presented. The reusability is perfectly befitting the current global societal trend of reduction of waste materials and promotion of environment-friendly new technologies. The proposed resonator has a CELC geometry and possesses higher sensitivity compared to various other sensor designs of comparable dimensions. The overall sensitivity of the sensor is improved compared to the conventional sensors by extending the metallic walls around the active region into the substrate for increased capacitance and concentrated field intensity. The sensor is able to detect the changes in the glucose concentration of aqueous solutions and real blood samples. The test samples are placed in disposable glass capillaries, isolating the samples from external contamination and thus making the sensor reusable. The most prominent advantage of the proposed scheme is the reusability of the sensor as a result of which the detection becomes extremely fast as the channels and the sensor do not require to be cleaned after each measurement. The method is quite economical and has abundant scope in the characterization of various other biological as well as chemical samples. The study can contribute more towards microwave-based blood glucose monitoring discipline by exploring the possibilities of characterization of healthy blood samples within normal glucose levels.
